# Supplementary material for: Effect of Micronutrient Powder (MNP) with a Low-Dose of Iron on Hemoglobin and Iron Biomarkers, and Its Effect on Morbidities in Rural Bangladeshi Children Drinking Groundwater with a High-Level of Iron: A Randomized Controlled Trial
Source: Nutrients. 2019 Nov 13;11(11):2756. doi: 10.3390/nu11112756 (PMC6893643; doi:10.3390/nu11112756)
Supplement: Supplementary file 1 [file nutrients-11-02756-s001.pdf]

Supplementary Text 1: The sample profile of hemoglobin and iron status parameters

As per the requirement of the sample size to detect a non-inferior difference in the hemoglobin estimates between the groups, approximately 70% of the enrolled (n = 327) children were randomly selected for blood sample collection. This translated in 231 for hemoglobin and 230 for ferritin, CRP, and AGP. Out of 231 samples of hemoglobin, 111 and 120 samples were in the standard MNP and the low-iron MNP groups respectively. Out of 230 samples for ferritin, CRP and AGP, 111 and 119 samples were in the respective treatment groups. Over the intervention period, 9 samples were excluded (7 refused, 1 migrated, and 1 diagnosed with a congenital disorder of colon), leaving 221 samples at end-point. For hemoglobin, ferritin, CRP, and AGP, out of these 221 samples, 106 and 115 samples belonged to the standard MNP and the low-iron MNP groups respectively. Serum transferrin receptor (sTfR) was sub sampled in 106 samples, paired over baseline and end-point. Of this, 47 and 59 samples belonged to the standard MNP and the low iron MNP groups, respectively. At baseline, 222 samples were tested for congenital hemoglobin disorders; 107 in the standard and 115 in the low-iron MNP groups, respectively.

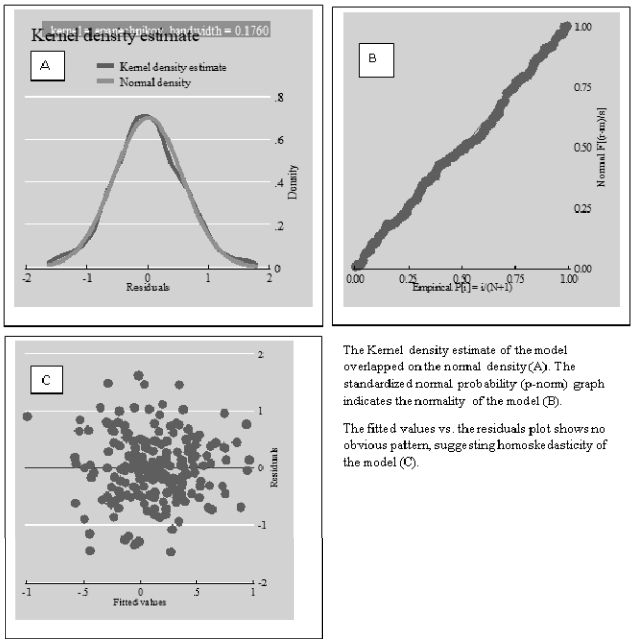

The Kernel density estimate of the model overlapped on the normal density (A). The standardized normal probability (p-norm) graph indicates the normality of the model (B).

The fitted values vs. the residuals plot shows no obvious pattern, suggesting homoskedasticity of the model (C).

Figure S1: Assessing normality and homoskedasticity of the multivariable model for the treatment effect of the low-iron MNP.

Table S1: Changes in hemoglobin and iron status markers, and comparative treatment effect of the low-iron MNP.

Variable	Standard MNP		Low-Iron MNP		β	95% CI	*p*-Value
	**Mean**	**SE**	**Mean**	**SE**	**(Robust SE)**		
	Hemoglobin (g/dL)				Treatment effect [*,‡] (Reference: standard MNP group)		
Baseline	12.25 [§]	0.07	12.38	0.07			
End line	12.46 [§ǀ]	0.07	12.39	0.07			
Change [†]	0.23	0.06	0.03	0.06	−0.14 (0.08)	-0.30, 0.018	0.08
	Serum ferritin (ng/mL)				Treatment effect [*,‡] (Reference: standard MNP group)		
Baseline	68.8 [§]	3.75	62.42	2.70			
End line	72.04 [§]	3.25	69.68 [ǀ]	2.95			
Change [†]	5.09	2.79	6.80	2.10	−0.4 (3.25)	−6.77, 5.94	0.90
	Serum TfR (μg/mL)				Treatment effect [*,‡] (Reference: standard MNP group)		
Baseline	3.99 [§]	0.14	3.89	0.13			
End line	3.93 [§]	0.14	3.64 [ǀ]	0.11			
Change [†]	−0.06	0.08	−0.20	0.09	−0.20 (0.12)	−0.44, 0.03	0.09

* Generalized Linear Model was used. † Changes in hemoglobin, ferritin, and sTfR between end-point and baseline were the dependent variables; treatment group was the independent variable; the covariates for adjustment were: age, gender, thalassemia status, mother's education; possession of cultivable lands; household food insecurity; spends on purchasing food; height-for-age Z score; baseline iron status markers depending on the type of the biomarkers analyzed; baseline morbidities, e.g., suffering from loose stools; baseline intake of dietary and groundwater iron; and the intake of iron from MNP. [‡] Per-protocol principle is applied. [§] The estimates were not statistically different from the corresponding estimates of the other treatment group ($p > 0.05$). [ǀ] The estimates were significantly different from the corresponding baseline estimates ($p < 0.05$)

Table S2: Contribution of iron from dietary, groundwater and MNP by the treatment groups.

	Source of Fe (mg/day)			
**Treatment Group**	**Diet [†]**	**Groundwater [‡]**	**MNP [§]**	**Total [ǀ]**
		**Mean ± SD**		
MNP	3.11 ± 1.74	4.78 ± 5.07	10.56 ± 2.66	18.25 ± 6.8 *
Low-iron MNP	3.39 ± 2.49	4.80 ± 5.86	4.3 ± 50.9	12.37 ± 6.3

* $p < 0.001$ for group difference. [†] The daily dietary intake of Fe was measured by a seven-day SQFFQ. [‡] The intake of iron from groundwater was estimated by multiplying the amount of water intake in the preceding 24 h with the concentration of iron in the groundwater (i.e., tube well) used for drinking. [§] The daily average intake of iron from the MNP was calculated as the number of sachets of MNP consumed over the

two months of the intervention, times the dose of the iron present in the sachet, divided by 60. | The daily total intake of iron was calculated by combining the intakes from all the sources.

Table S3: Consumption profile of MNPs

**Standard MNP (*n* = 164)**			**Low-Iron MNP (*n* = 163)**			**All (*n* = 327)**		
**Mean ± SD***	**Median**	**%†**	**Mean ± SD**	**Median**	**%¹**	**Mean ± SD**	**Median**	**%†**
50.68 ± 12.7	54.0	84.46	52.22 ± 11.0	55.0	87.0	51.45 ± 11.9	54.1	85.75

* $p > 0.05$ for the group difference. † Proportion to the allocated number of sachets ($n = 60$).

Supplementary Text 2: An assessment of potential bioavailable iron from all sources in children taking the low-iron MNP

Worwood [49] et al. has calculated the absorption of iron from iron-rich natural water. Estimated absorptions were 40% for the iron-depleted subjects (ferritin < 10 ng/ml); ~10% for the subjects with ferritin ~200ng/mL. The average rate of absorption was 23%. The rate of absorption and serum ferritin had a high negative correlation ($r = -0.78$). We did not study the absorption of iron from groundwater. The iron in groundwater in Bangladesh largely exists as the ferrous form (99%) when freshly extracted, which is readily bioavailable [47]. Since, the infection-adjusted ferritin concentration in our participants was ~65 ng/mL, which was neither deficient nor very high, we conservatively assumed the absorption rate of iron could be within the lowest (10%) and the average values (23%) reported in Worwood's study.

At 10% absorption of iron from groundwater (considering the actual intakes of iron as presented in Table S2), the potential absorption of iron from groundwater = 4.8 mg * 0.1 = 0.48 mg/day. From diet = 3.39 mg* 0.05 = 0.17 mg/day (considering 5% absorption as the diet is cereal based). From the low-iron MNP = 4.35 mg* 0.046 = 0.20 mg/day (considering 4.6% absorption from MNP [47]). Thus, the total amount of potentially bioavailable iron from all sources = 0.48 + 0.17 + 0.20 mg = 0.85 mg/day.

At 23% absorption of iron from groundwater (considering the actual intakes of iron as presented in Table S2), the potential absorption of iron from groundwater = 4.8 mg* 0.23 = 1.1 mg/day. From diet = 3.39 mg* 0.05 = 0.17 mg/day (considering 5% absorption as the diet is cereal based). From the low-iron MNP = 4.35 mg* 0.046 = 0.20 mg/day (Considering 4.6% absorption from MNP [47]). Thus, the total amount of potentially bioavailable iron from all sources = 1.1 + 0.17 + 0.20 = 1.47 mg/day.
